# Colistin Resistant *mcr* Genes Prevalence in Livestock Animals (Swine, Bovine, Poultry) from a Multinational Perspective. A Systematic Review

**DOI:** 10.3390/vetsci8110265

**Published:** 2021-11-04

**Authors:** George Valiakos, Ioanna Kapna

**Affiliations:** Faculty of Veterinary Science, University of Thessaly, 43100 Karditsa, Greece; ikapna@uth.gr

**Keywords:** bovine, cattle, chicken, colistin resistance, *mcr*, poultry, swine

## Abstract

The objective of this review is to collect and present the results of relevant studies on an international level, on the subject of colistin resistance due to *mcr* genes prevalence in livestock animals. After a literature search, and using PRISMA guidelines principles, a total of 40 swine, 16 bovine and 31 poultry studies were collected concerning *mcr*-1 gene; five swine, three bovine and three poultry studies referred to *mcr*-2 gene; eight swine, one bovine, two poultry studies were about *mcr*-3 gene; six swine, one bovine and one poultry manuscript studied *mcr*-4 gene; five swine manuscripts studied *mcr*-5 gene; one swine manuscript was about *mcr*-6, *mcr*-7, *mcr*-8, *mcr*-9 genes and one poultry study about *mcr*-10 gene was found. Information about colistin resistance in bacteria derived from animals and animal product foods is still considered limited and that should be continually enhanced; most of the information about clinical isolates are relative to enteropathogens *Escherichia coli* and *Salmonella* spp. This review demonstrates the widespread dispersion of *mcr* genes to livestock animals, indicating the need to further increase measures to control this important threat for public health issue.

## 1. Introduction

Colistin as an antimicrobial agent belongs to the polymyxin antibiotic class and it is produced by *Paenibacillus polymyxa*, which is a Gram-positive bacterium. This antibiotic class consists of five polymyxins, A, B, C, D, and E from which polymyxin E (colistin) and polymyxin B are used clinically [1,2,3]. Colistin is a commonly used drug in the animal field for two main reasons; colistin treats infections caused by Enterobacteriaceae and it is a growth promoter and a protective agent. The class of polymyxins, where both colistin and polymyxin B belong to, is one of the primary classes of antibiotics against most Gram-negative bacteria [4,5].

Polymyxins antibacterial spectrum is narrow, mainly against common Gram-negative bacteria. They are active against most of the bacteria of Enterobacteriaceae family, including *Escherichia coli*, *Enterobacter* spp., *Klebsiella* spp., *Citrobacter* spp., *Salmonella* spp., and *Shigella* spp. Polymyxins activity is also effective against common non fermentative Gram-negative bacteria, such as *Acinetobacter baumannii*, *Pseudomonas aeruginosa*, and *Stenotropomonas maltophilia* [6,7,8]. Furthermore, due to observation of a post antibiotic effect against *K. pneumoniae*, *P. aeruginosa*, and *A. baumannii* [9,10,11], polymyxins are used as a last-resort treatment option against them [12,13,14].

Colistin was discovered in 1947 and was used in Europe and Japan during the 1950s [15]. However, the use of polymyxins was reconsidered in the 1970s, because of the toxicity and especially nephrotoxicity researchers observed. Therefore, they were then replaced by more active and less toxic antibiotics, such as aminoglycosides, quinolones, and β-lactams. In addition, the use of colistin was restricted to ophthalmic and topical uses for 20 years and systemic or nebulized colistin was used only in case of cystic fibrosis [4,16].

It is true that over the last several years there is a lack of new antibacterial chemical entities and Gram-negative bacteria develop a rapid resistance to current antibiotics. For that reason, colistin was overused in both human and veterinary medicine, also as a growth agent or protector [5,17,18,19,20]. Colistin is used as therapy, as protector, and even as a growth promoter especially in swine, in some countries [21,22]. The use of colistin and tyrosine as supplementation helped to the increase of body weight and the amount of feed intake in broilers [23]. Studies reporting colistin use from various countries demonstrated that China was the highest user of colistin in agriculture but in 2017, colistin was banned to be used as a growth factor for livestock [24].

An important contributor to the emergence of antibiotic-resistant bacteria is suspected to be the extensive use of antibiotics for animal growth promotion and there is a global concern not to disseminate them from farms into the wider environment [25,26,27].

The latest recommendation of European Medicines Agency (E.M.A.) advises that: (1) European Union (E.U.) Member States should sale colistin in minimum amount as possible for use in animals, so that to achieve a 65% reduction in E.U.-wide sales, (2) colistin be added to a more critical category of medicines, reserved for treating clinical conditions only if there are no effective alternative treatments. Colistin methanesulfonate sodium, for parenteral use, must be administered carefully, as there is a new interest in use of colistin to infections caused by multidrug-resistant bacteria and there are increasing rates of colistin resistance currently observed [4].

China remains first worldwide in use of colistin in agriculture with 11.942 tons per year by the end of 2015. China will have a 4.75% average annual increase of colistin use as expansion and intensification of animal husbandry is observed and for that reason the annual quantity use will be 16.500 tons by end of 2021 [28]. Colistin is no longer used as a growth promoter in China, since April 2017, because of its importance to human clinical chemotherapy [24]. This policy is agreed with the One Health framework [29]. The ‘One Health’ approach was defined by the American Veterinary Medical Association as the collaborative efforts of multiple disciplines working locally, nationally, and globally to attain optimal health for people, animals, and our environment [30]. Interactions similar to this should take place at different levels, for example when there is a zoonotic infectious disease outbreak to manage in the field or a joint research program to develop [31].

The first plasmid found to carry a mobile colistin resistance (*mcr*) gene, in July 2013, is the pHNSHP45 plasmid (GenBank access number: KP347127), which was called *mcr*-1 and recovered from *E. coli*, isolated from a Shanghaian pig. The gene enables the bacterium to be colistin and polymyxin B resistant [28].

This transferable resistance gene was supposed to originate from livestock populations as we know that colistin is rarely used in humans because of nephrotoxicity and neurotoxicity and most *mcr*-1–positive strains were isolated from livestock samples [1]. Until now, various genes encoding colistin resistance have been identified (*mcr*-1, *mcr*-2, *mcr*-3, *mcr*-4, *mcr*-5, *mcr*-6, *mcr*-7, *mcr*-8, *mcr*-9, and *mcr*-10) [32,33,34,35,36,37,38,39,40,41]. All *mcr*-genes found are similar to one another, homologous, and they work under the same mechanism to provide resistance to colistin [17]. Two genes, *mcr-1* and *mcr-3* have spread around the world [42] and *mcr-2* was the only one detected in Europe [43]. Currently, 22 functional genetic variants of *mcr-1* detected (from *mcr-1.1 to mcr-1.22*) have been uploaded to NCBI GenBank. These variants differ from *mcr-1* by one or a few amino acids. In addition, they all have a high nucleotide and amino acid identity (∼99%), and thus confer a similar effect on colistin resistance [44,45,46,47].

Other *mcr* genes are also divided in variants. The *mcr-2* gene (*mcr-2.1*, *mcr-2.2*, *mcr-2.3*) was detected in *E. coli* from calves and piglets in Belgium [38], whereas *mcr-3* (from *mcr-3.1* to *mcr-3.30*) in *E. coli* from pigs in China [41]. The *mcr-4* gene (from *mcr*-4.1 to *mcr-4.6*) was found in *E. coli* and *Salmonella enterica* serovar Typhimurium from pigs in Italy, Spain and Belgium [40] and *mcr-5* (from *mcr-5.1*, to *mcr-5.4*) in *Salmonella* Paratyphi B from poultry in Germany [39]. The *mcr-6.1* gene was recently noted and deposited into GenBank (NG_055781) [32]. In addition, more recently, *mcr-7* was described in *K. pneumoniae* strains from chickens, in China [34] and *mcr-8* (*mcr-8.1* to *mcr-8.4*) was recovered in New Delhi metallo-β-lactamase (NDM) producing *K. pneumoniae* not only from food-producing animals but human clinical samples as well [33].

A new *mcr* gene (*mcr*-9) was described by Carroll et al. in 2019 [35]. Carroll and his team were conducting the routine checking of the *Salmonella* genome sequences for antimicrobial resistance genes while they identified this new gene in a *Salmonella enterica* serotype Typhimurium (*S.* Typhimurium) strain isolated from a human patient in Washington State in 2010. However, currently the *mcr-9* gene is not associated with colistin resistance in the United States [48]. A year after, Wang et al. [36] described *mcr-10*, another brand-new *mcr* gene, on the IncFIA plasmid of *Enterobacter roggenkampii* isolate.

Multiple studies have found that the *mcr*-1 gene spreads rapidly in animals, travelers, foodstuffs, and the human environment around the world [19,46,49]. The *mcr* gene was identified in over 11 species of Enterobacteriaceae, with bacterial samples deriving from five different continents, in a total of forty countries, taken from the environment, alimentary products, living animals (e.g., pig, poultry and cattle) and humans [50,51,52].

On the other hand, *E. coli* owes mostly its colistin resistance ability to the existence of the *mcr-1* and *mcr-2* genes, since these two were prevalent in isolates in comparison to the *mcr-3* gene [53,54].

The resistance is due to the ability of the *mcr-1* gene to encode a phosphoethanolamine transferase that modifies lipid A, reducing its attraction for colistin [55]. In other words, specific resistance to colistin by the plasmid-mediated *mcr-1* gene is facilitated by encoding a phosphoethanolamine transferase, which modifies lipid A using a phosphoethanolamine (PEP) group, preventing interaction between colistin and lipid A [12,56,57,58]. The bactericidal activity of colistin causes cell membrane disruption, as an electrostatic interaction occurs between colistin and the lipid A portion of bacterial LPS. The resistance to colistin is immediate, albeit non-specific and mediated through transcriptional up-regulation of drug efflux pumps. A previous research study indicated that colistin resistance in *E. coli* and *Salmonella* isolates, is probably due to a mutation in the two-component PhoP-PhoQ and/or PmrA-PmrB systems [59,60]. Consequently, these mutations cause structural modifications in the lipid A subunit, reducing electrostatic interactions between the positively charged amino groups of colistin and negatively charged phosphate groups of the lipid A subunit, preventing disruption of the cell membrane [61,62,63].

Furthermore, researchers found a perceivable resistance to colistin in some species of the Enterobacteriaceae family. This resistance caused by the modification of 4′-phosphoethanolamine (PEA) of lipid A on the LPS [37,64].

The objective of this review is to present the results of relevant studies on an international level, on the subject of colistin resistance due to *mcr* genes prevalence.

## 2. Materials and Methods

**Figure 1:**
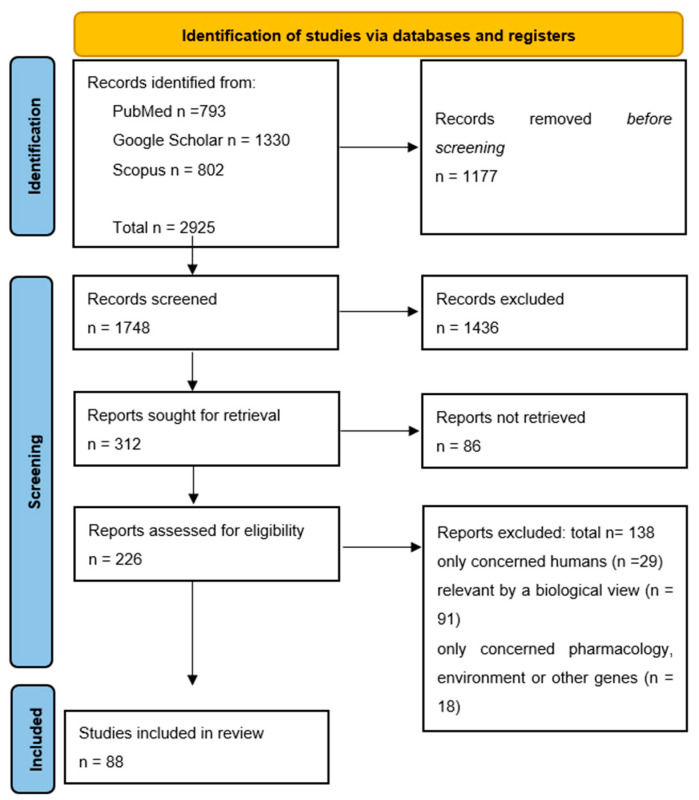
Identification of studies regarding mcr genes in livestock animas via databases using PRISMA guidelines. From: Page MJ, McKenzie JE, Bossuyt PM, Boutron I, Hoffmann TC, Mulrow CD, et al. The PRISMA 2020 Statement: An Updated Guideline for Reporting Systematic Reviews. BMJ 2021;372:n71. doi:10.1136/bmj.n71.

This study attempted to define certain criteria that will enable a systematic search of data to fulfill its objectives. We followed the Preferred Reporting Items for Systematic Reviews (PRISMA) guidelines (Figure 1). Reviews with the subject of colistin resistant *mcr* genes prevalence in livestock animals worldwide were searched in the following databases: Google Scholar, Scopus, PubMed. In these databases, 2925 studies were collected, their findings analyzed, from 2016 to 2021, containing the keywords colistin, resistance, *mcr*, swine, bovine, cattle, poultry, pigs, chickens, or a combination of them.

The selected studies were published in peer-reviewed journals, websites of organizations, books, and dissertations mainly in the English language. A first screening was based on the titles. At first, we excluded duplicates as well as studies related to the economic impact, diagnostic tests, human medicine, vaccine, genetics, prediction models, nutritional studies reported to vegetables, water or environment and other studies referred to wild animals which were not related with the factors we were looking for. Similarly, we excluded studies in species other than cattle, poultry, swine. The second selection phase examined the abstracts in order to identify fully and independently reviewed studies, to assess their relevance according to the information that were searched. Generic information was collected from each article, such as the author, year of publication, country where the study was conducted, its design and unit of interest and number of subjects.

Specifically, a total of 2925 manuscripts were found: 793 in PubMed, 1330 in Google Scholar, 802 in Scopus. A total of 1177 publications were excluded based on title irrelevance, were duplicates, did not provide an abstract or were written in different unknown language. Subsequently, of the remaining 1748 articles whose abstracts were examined, 1436 were rejected because their abstracts were irrelevant to the scopus of this review, according to the criteria referred previously. For these reasons, 312 manuscripts remained and 86 could not be retrieved. Therefore, 226 studies left to be examined and among them we found and rejected 138 with the reason that they only concerned humans, were relevant to the subject by a biological view, only concerned pharmacology, the environment, or other genes. Finally, 88 manuscripts were used in this review.

The information extracted from each relevant article is thoroughly collated and presented. The findings were organized in separate tables, one for each species (Table 1, Table 2 and Table 3). Each table contains information concerning the country, the bacteria species that were studied, the types of *mcr* genes that were searched for, the total number of sampled isolates, the total number of phenotypically colistin resistant isolates, the total number of *mcr* genes detected, the types of *mcr genes* detected, the percentage of *mcr* genes prevalence in samples and the relevant citation.

## 3. Results

### 3.1. Swine

Swine studies had to do with samples collected from 15 countries; these studies occurred in China (15), Spain (4), Germany (3), Japan (3), England (2), Italy (2), Portugal (2), Vietnam (2), Belgium (2), Thailand (2) while France, Brazil, Canada, South Korea, Switzerland have one each. There is also a study that used samples from various European countries. Most of these studies use *E. coli* bacteria (36), with other bacteria following, such as *Salmonella* spp. (7), *Klebsiella* spp. (3), *Kluyvera* spp. (1), *Aeromonas* spp. (2), *Moraxella* spp. (2) and various genera of the Enterobacteriaceae family (6). Worldwide, the *mcr*-1 gene was indicated to be the most prevalent mobile colistin resistance gene [65,66]. The findings are organized in Table 1.

#### 3.1.1. China

In a study having the aim to characterize multidrug-resistant Shiga toxin-producing *Escherichia coli* (STEC) the *mcr-1* gene was also found on plasmids isolated from pigs in Guizhou province of China [67]. This study, in which 10 colistin-resistant isolates were identified and found to contain the *mcr-1* gene, using PCR method to identify them, is a follow-up to an earlier investigation.

Three more provinces of China were investigated [68]. Among hundreds of bacteria isolated from porcine tissues, only 16 strains were found to be colistin resistant. PCR showed that six of them were positive for the *mcr-1* gene. All six *mcr-1* positive *E. coli* samples, originating from four different provinces increase the risk for co-existence of *mcr-1* and other antibiotic resistance genes.

As shown from data presented by Wang and collaborators [69], in 2016, *mcr-1* colistin resistance in gut microbiota is more complicated, a fact that constitutes a potential threat to public health and clinical treatment. Amongst 1026 pieces of *Escherichia coli* isolates which were collected from three different pig farms, 302 isolates were determined to be positive for the *mcr-1* gene (30%, 302/1026).

Moreover, the *mcr-1* gene could be prevalent in other zoonotic pathogens as well, such as *Salmonella* bacteria. Scientists detected the colistin resistance gene *mcr-1* in *Salmonella* isolates from both humans and diarrheic animals and the resistance gene was detected on different plasmids. Antimicrobial susceptibility testing was conducted on various *Salmonella* isolates from human and animal sources. Among them, 279 isolates derived from pigs, identifying 20 colistin resistant isolates of which seven *mcr-1* gene carrying isolates from seven pigs. The *mcr-1* carrying isolates from animals proved to be more resistant than the human isolates [70].

Colistin had been used in animals as a therapeutic drug and feed additive since the early 1980s. The study of Li et al. [71], in 2018, was conducted in order to evaluate the occurrence of *mcr-1* in *E. coli* isolates from healthy or diseased pigs, in Henan, province of China. A total of 306 *E. coli* isolates were retrieved, of which 78 were *mcr-1* positive. Of these, 46 belonged to 102 isolates derived from diseased pigs and the rest 32 were obtained from 204 isolates derived from healthy pigs, demonstrating a significant increase of *mcr-1* presence in diseased animals.

Aiming to augment the general understanding of colistin resistance genes, another study took place the same year [72], collecting and analyzing a total of 600 fecal samples from 60 swine farms in 18 provinces of China. All *E. coli* colistin-resistant colonies were identified positive to the *mcr-1* gene, detecting *mcr-1* in an extremely high rate (457/600, 76.2%), with positive rates ranging from 45.0–100% among the provinces.

Since *mcr-4* and *mcr-5* genes were identified quite recently, a study [73] attempted to evaluate the prevalence of *mcr-1* and *mcr-2* genes in comparison to the newly discovered ones, by employing the PCR method on samples derived from both edges of the digestive system. The *mcr-1* specific PCR detected the gene in 83.6% of anal and 79.0% of nasal swabs, the *mcr-2* prevalence in pigs was 56.3% (nasal: 58.4%, anal: 23.0%) and the *mcr-3* was detected in 8.4% of samples. Dual positivity was identified in 730 pigs for *mcr-1* and *mcr-2*, 267 pigs for *mcr-1* and *mcr-3*, and 177 pigs for *mcr-2* and *mcr-3*. As a result, it seems that the *mcr-1*, *mcr-2,* and *mcr-3* are relatively common and widespread in food producing animals of China.

During 2016 and 2017, fecal animal samples were collected from 32 swine farms in 13 provinces, and the 174 fecal samples of farmers were collected from 16 farms in nine provinces, to study *mcr-3*-mediated colistin resistance. Of them, 49 samples were found positive in *mcr-3* (49/6497, 0.75%), verified by PCR and Sanger sequencing; six of them derived from farmers and the other 43 were from swine-derived samples. Furthermore, no *mcr-3* samples were identified positive from patients or other healthy individuals. In addition, 14.02% (911/6497) samples were *mcr-1*-positive. Randomly eight isolates out of 49 *mcr-3*-positive samples were chosen for microbial analyses and all had resistance to colistin and polymyxin B. In addition, it was found that phylogenetically, *mcr-3* is widely different from *mcr-1*. It appears that *mcr-3* and its variants might have a parallel evolutionary path to *mcr-1*, but the chromosomal progenitor has not yet been identified [74].

In another study [75], researchers investigated household pig samples and found a total amount of 17% (71/417) of *E. coli* samples positive for *mcr-1* and 1.2% (5/417) of samples positive for *mcr-3*. *Mcr-2*, *mcr-4* and *mcr-5* were not detected in this particular case. This report was the first detecting *mcr-3* in backyard pig husbandry revealing that *mcr* genes are not restricted to commercial pig farms, but also detected in small-scale backyard holdings.

Regarding *mcr-4* and *mcr-5* gene prevalence in 2018, Chen et al. [76] investigated their presence in China, by employing PCR. In swabs collected from swine, the prevalence of the *mcr-4* was 41.4% (642/1552) and *mcr*-5 33.1% (514/1552). Both the *mcr-4* and *mcr-5* were detected in 18.3% (266/1454) of the pigs. It was also indicated that the prevalence of the *mcr-4* and *mcr-5* genes were significantly higher in the nasal/oropharyngeal swabs than in the anal/cloacal swabs.

The first report of the *mcr-5* gene isolated from *Aeromonas hydrophila* was published by Ma et al. in 2018 [77]. The gene was isolated from backyard pigs in rural areas of China and was characterized. *Mcr-5* differs from *mcr-1*, *mcr-2*, *mcr-3,* and *mcr-4*, having only 34–36% amino acid sequence identity to the other four proteins. Bacteria of the genus *Aeromonas*, especially *A. hydrophila*, *A. caviae* and *A. veronii*, is common to cause diarrheal diseases and wound infections in both humans and animals, and they appear widespread in aquatic environments. Only one *mcr-5* positive *A. hydrophila* isolate was detected, while *mcr* genes were not detected in *E. coli*, A. *veronii* and *A. caviae* strains isolated.

In the aftermath of the colistin restrictions in China, the study of Xia et al. [78] attempted to investigate, on one hand, the influence of said restrictions on the prevalence of the *mcr-1* gene, while on the other hand, to determine whether decrease of the *mcr-1* gene spread on the environment could be achieved if swine waste is treated in specially designed for this purpose facilities in farms. This was the first data showing the reduction of abundance of *mcr-1* and the association of colistin residue before and after the obligatory withdrawal of colistin as growth promoter in China.

A similar study [79] at the same year sought to determine the prevalence of colistin resistance due to *mcr* genes and the molecular epidemiology of *mcr-1* and *mcr-2* among farms in Jiangsu Province. The *mcr-1* gene was detected in colistin-resistant *E. coli* colonies isolated from pigs of all ages (68.86%) same as the *mcr-2* (46.82%) and both *mcr-1* and *mcr-2* were isolated in 20% of the total pig population. It is possible that the increasing colistin use in fodder in recent years is the reason for the high prevalence of colistin resistance in animals. Colistin resistance in *E. coli* is promoted by both *mcr*-independent and *mcr*-dependent mechanisms. This study provided new data about colistin resistance prevalence worldwide. The dominant mechanism in *E. coli* was the *mcr*-dependent mechanism and there is a high frequency of colistin resistant *E. coli* in food animals of all ages. It was also found that the reservoir of resistant strains were older and adult animals.

Another study focused on the presence of mobile colistin gene in swine production environment and analyzed the genomic environment of positive isolates to the new colistin resistant gene by subjection to the whole genome sequencing (WGS). In total, 33 *mcr-1* and/or *mcr-3* positive isolates were identified, but since none of the other six *mcr* genes were discovered, the study concluded that only the *mcr-1* and *mcr-3* genes were widely spread in swine farms. In addition, two plasmids which carried different *mcr* genes and were co-transferred, were recognized [80].

Two years later, Shen et al. [81] investigated the subsequent changes in *mcr-1* prevalence, and the genomic epidemiology of *mcr-1* positive *E. coli* before and after the prohibition of colistin use (October–December 2017 and 2018, respectively). China banned the use of colistin in animal feed from 1st May in 2017. After the ban, *mcr-1* prevalence decreased significantly in national pig farms, from 45% (308/684 samples) in 2016, to 19,4% (274/1416) in 2018.

#### 3.1.2. Thailand

The aim of a study [82] was to examine the prevalence and genetic characteristics of ESBL-production and colistin resistance in the bacteria of *Salmonella* and *E. coli*. Isolates from pigs and pork meat of the border area among Thailand, Cambodia, Lao PDR, and Myanmar were examined. Specifically, in October 2017 and March 2018, a total of 463 *Salmonella* and 767 *E. coli* isolates were collected from pigs in 441 slaughterhouses using rectal swabs and other isolates were collected from pork in 368 retail markets. Colistin-resistance rate in *E. coli* (10.4%) was significantly higher than *Salmonella* (2.6%). The *mcr-1* gene was detected in *Salmonella* (*n* = 12) and *E. coli* (*n* = 68). The *mcr*-1/blaCTX-M-55 was observed in one *Salmonella* and *mcr*-3/blaCTX-M-55 co-concurrence was observed in three *E. coli* isolates.

A study in Thailand during the years 2014–2017 aimed to investigate patterns of antimicrobial resistance, to compare the proportions of multidrug resistance and to detect the existence of perceivable colistin resistance (*mcr*) genes, *mcr*-1, *mcr*-2, and *mcr*-3, among *Salmonella* isolates collected from pork in slaughterhouses and retails. The results demonstrated five multi-drug resistant (MDR) *Salmonella* isolates carrying the *mcr*-3 gene, while only four isolates (1.33%) displayed colistin resistance phenotype. In conclusion, this Thailand study was the first report in the country that found *mcr*-3 positive MDR *Salmonella* isolates collected from pork [83].

#### 3.1.3. Spain

Until 2016 only *mcr-1*, *mcr-3*, and *mcr-4* have been detected in Spain [40,53,84] and two years after the Spanish Agency of Medicines and Medical Devices (A.E.M.P.S.) [85] established a voluntary strategic plan, which was called “Programa Reduce Colistina” within the Spanish Plan against antibiotic resistance (PRAN) to reduce colistin use in pigs [86]. A total of 70% of Spanish pig production companies joined the program, representing 80% of Spanish pig production [85]. It aimed to early indicate the impact of these recommendations and how the elimination in antibiotic use can influence the levels of colistin resistance in Spanish pigs. A similar trend was observed in results, published by the A.E.M.P.S., of total veterinary sales of colistin in Spain [85]. Colistin use in Spain was common practice until 2011 when restrictive legislation on it was enforced (from 29.38 mg/PCU in 2012 to 21.4 6 mg/PCU in 2013) [85]. A.E.M.P.S. results about reduction are parallel to the observed decline in colistin-resistant bacteria in another study, a mentioned by Miguela-Villoldo and collaborators in 2019 [87].

A 2020 study aimed to identify antibiotic resistance patterns and genes in Spanish pigs during the last twenty years. Susceptibility to six antibiotics commonly used in pig production was tested in quality and quantity in 200 strains of *E. coli* which had been isolated from clinical cases of diarrhea in neonatal and post-weaned piglets, between 1999 and 2018. Results showed resistance to colistin. The resistance values peak between 2011 and 2014 (17.5% of the strains). The *mcr-4* was the most frequent colistin resistance gene, in 13% (26/200) of strains. Both *mcr-1* and *mcr-5* were detected in 7% (14/200) and 3% (6/200) of the strains respectively, while *mcr-2* and *mcr-3* genes were not detected in any of the studied strains. The 97% reduction in the use of colistin in swine from 2015 to 2018 in Spain might be related with the following significant decrease in colistin resistance reported by the A.E.M.P.S. in 2019 [88].

Migura-Garcia and collaborators in 2020 studied the *mcr*-mediated resistance plasmids in *E. coli* isolated from animals in Spain. Only a total of 14 *mcr-1* and one *mcr-4.2* gene carrying isolates were detected, without any other *mcr*-variants being found. All measurements used the PCR method for the detection of the *mcr-1, mcr-2, mcr-3*, *mcr-4* and *mcr-5* genes [89], and no other mechanisms of resistance were tested. It is worth mentioning that a variety of resistant genes was detected for different families of antimicrobials (aminoglycosides, beta-lactams, tetracycline, sulfonamides, phenicol, and trimethoprim) in *mcr* gene carrying plasmids from pigs. These genes could possibly affect positively bacterial resistance in colistin, despite the decrease in its application in swine farms [90].

#### 3.1.4. Germany

A German study attempted to indicate the presence of the *mcr-1* gene in a total of 577 whole genome sequences of isolates obtained from different sources (human, animal, and environmental) since 2009 in Germany. The *mcr-1* gene was detected in four *Escherichia coli* isolates, three originating from swine. The isolate V163 was obtained in 2010, indicating that the existence of transmissible colistin resistance in German animals is an older occurrence. The *mcr-1* gene was detected on different classes of plasmids in isolates of various sequence types. This suggests that multiple pathways for horizontal transmission of this resistance exist. This study suggests that nowadays we already have a problem with untreatable infections as every colistin-resistant isolate of the study is also resistant to either third-generation cephalosporins or to carbapenems [91]. To obtain an overview of the prevalence of *mcr-1* and *mcr-2* colistin resistance genes in German pig farms, a study included 436 boot swabs and pooled faecal samples collected from 58 farms. The presence of *mcr-1* was detected in 43 *Escherichia coli* isolates from 15 farms. This indicated that the *mcr-1* gene was present in 9.9% of the analyzed samples and 25.9% of the investigated pig farms. In contrast, none of the tested samples was found positive for *mcr-2* [92].

Another study investigated the prevalence of extended-spectrum β-lactamase (ESBL) and AmpC-producing Enterobacteriaceae in German fattening pigs and explored potential risk factors. The aim was to investigate factors associated with the occurrence of *mcr-1*. Overall, in 12 of 48 farms, at least one sample was found positive to *mcr-1* gene isolated from *E. coli*, resulting in 25% of positive pig farms [93].

#### 3.1.5. Japan

Since July 2018, the aim in Japan is to reduce the development of colistin-resistant bacteria and the spread of their plasmid-based *mcr* genes. If the use of colistin as feed additive in animals was banned, the effects may be minimal on humans. In order to evaluate the effect of colistin as feed additive, five pigs were examined from birth to finishing in a farm. For that, *E. coli* samples derived from pig feces, in distinct three farm fields, were characterized before and after the use of colistin as a feed additive to the livestock. The study showed that colistin resistance (due to *mcr*-1 gene) in *E. coli* isolated from these five pigs increased during the colistin administration period and decreased immediately after its end. In addition, despite this decrease, 12 months after the ban of colistin use the researchers still detected, in farm fields, colistin resistance and *mcr*-1-positive *E. coli* [94].

In total, 684 strains were investigated in Japan for susceptibility to colistin and for *mcr-1* existence. Swine-pathogenic *E. coli* strains were isolated from farm animals, but not food products and *mcr-1* gene was detected in 90 (13%) strains, while the MICs for these *mcr-1*–positive strains ranged from 8 to 128 mg/mL. These results suggest that a great proportion of swine-pathogenic *E. coli* in Japan is resistant to colistin, that *mcr-1* has already a wide prevalence and that there is a similarity between the level of colistin resistance mediated by *mcr-1* and the level of resistance mediated by *mcr-1*–independent mechanisms [95].

Another review clarified the prevalence of colistin-resistant *E. coli* and plasmid-mediated colistin resistance genes isolated from various sources such as diseased pigs, healthy pigs and humans in Japan. Among the 120 isolates extracted from diseased pigs, the *mcr-1* gene was detected in 36 isolates (30%), *mcr-3* in 10 isolates (8.3%) and *mcr-5* genes in 34 isolates (28.3%). According to these results, spreading of *mcr-1*-harbouring plasmids is the reason for the high *mcr-1* prevalence among diseased pigs. Coexistence of *mcr-1* and *mcr-5* (4.2%; 5/120) was detected, but no other combinations were found. The *mcr-3* and *mcr-5* genes were highly distributed in diseased pigs [96].

#### 3.1.6. Great Britain

In a relevant study the objectives were to determine the prevalence of *mcr-1*-harbouring *E. coli* in swine material originating from Great Britain from 2013 to 2015 and characterize *mcr-1* plasmids. Using selective isolation, the occurrence of *mcr-1 E. coli* in the appendix from healthy pigs was 0.6% in 2015. The *mcr-1 E. coli* gene was also identified in isolates in 2015. All isolates committed before 2015 were negative. WGS analysis of four *mcr-1*-positive *E. coli* did not indicate other antimicrobial resistance (AMR) genes were linked to *mcr-1*-plasmid-bearing contigs, despite the existence of harboring multiple AMR genes. As a low number of *mcr-1*-positive *E. coli* isolates was identified during the survey, it was suggested that *mcr-1* is currently uncommon in *E. coli* derived from pigs within Great Britain [97].

A consequent study tried to determine the occurrence of not only *mcr-1* gene but also *mcr-2* gene in various Gram-negative bacteria isolated from healthy pigs. Variants of *mcr-1* and *mcr-2* were identified in *Moraxella* spp. isolated from appendix contents of healthy pigs collected from six farms. Other bacteria, such as *E. coli* from the same farms, were not detected harboring *mcr-1* or *mcr-2* genes. These results demonstrate themobilization of the *mcr*-pap2 unit from *Moraxella* via composite transposons which lead to a worldwide dissemination. The presence of *mcr*-pap2 from *Moraxella* isolates indicates they may be a reservoir for *mcr* genes [32].

#### 3.1.7. Portugal

Portugal is the fourth European country following Spain, Italy, and Croatia, concerning colistin sales levels (peaking in 2013) and the third country in consumption of colistin in food-producing animals [98,99].

A 2019 study aimed to investigate the presence of *mcr-1* and *mcr-2* genes in Enterobacteriaceae isolates from food-producing animals, animal feed and meat and its products during 2010–2015 in Portugal. This was the most extensive Portuguese study until then. Overall, *mcr-1*-like genes were detected in 100 colistin-resistant Enterobacteriaceae isolates. No positive isolates were found for the *mcr-2* gene. Regarding fattening swine and broilers, colistin resistance occurred at relatively lower levels, but similar, especially in swine samples, as reported in other studies. Indeed, the presence of colistin resistance gene in food indicates a potential public health danger, as it is located in mobile genetic elements that is possible to spread horizontally [47].

A year later, Fournier and collaborators determined the occurrence of colistin resistant and extended-spectrum ß-lactamase (ESBL), produced by Enterobacteriales from pigs at two farms in Portugal and evaluated possible correlations using different antibiotics. Ninety-three ESBL-producing isolates (62 *Escherichia coli*, 29 *Klebsiella pneumoniae*, one *Enterobacter aerogenes* and one *Enterobacter cloacae*) and 17 colistin-resistant isolates (12 *E. coli*, four *K.* and one *E. cloacae*) were identified. In total, from the 17 colistin resistant isolates that were collected from the first farm, 12 derived from *E. coli* and 3 from *K. pneumoniae* carried the *mcr-1* gene. This study exhibits high rates of ESBL (75%) and *mcr-1* gene (11%) production among enterobacterial isolates retrieved from pig farms in Portugal [100].

#### 3.1.8. Vietnam

The study of Nguyen provided information about antimicrobial levels of use, as well as phenotypic and genotypic resistance of critical importance antimicrobials among *E. coli* in different stages of pig and poultry production. *E. coli* isolates showed a high prevalence of resistance (>20%) to critically important antimicrobials, such as colistin. The *mcr*-1 gene is identified as responsible for colistin resistance and likely plays a major role in AMR to those compounds. Conjugation experiments identified a 63-kb plasmid, similar to that identified recently in China, as the potential carrier of the *mcr*-1 gene [101]. Two years later, another Vietnamese study investigated local foods in order to identify contamination of colistin-resistant bacteria. The 97% (60/62) of isolates, were colistin-resistant and *mcr*-1 gene was recovered, whereas *mcr*-3 was identified in 3% (2/62) of the isolates. Two strain plasmid analysis identified both plasmids harboring the *mcr*-3 gene. The prevalence of ESBL- or AmpC-producing *E. coli* in foods of Vietnam proved to rank higher, compared with those foods analyzed in Japan [102].

Nakayama and collaborators also reported that many Vietnamese farmers make use of colistin [103] and 4% of ESBL- or AmpC-producing *E. coli* showed colistin resistance. Among these 62 isolates, *mcr*-1 was found in 60 isolates and only two *mcr*-3 genes were recovered. Conjugation assays indicated that plasmids containing *mcr*-3 were transferable to recipient strains, and *mcr*-3 was carried by an IncFII plasmid. The presence of *mcr*-1 and *mcr*-3 gene in a number more than 20% of ESBL- or AmpC-producing *E. coli* demonstrated the necessity to study ESBL, AmpC and *mcr* genes at the same time [104].

#### 3.1.9. France

An experimental trial quantified the transmission of an *mcr*-1-positive *E. coli* strain from pigs. For the assessment of colistin use impact and better conditions in diverse fields, different experimental designs were implemented. More specifically, colistin given or not given to the inoculated or exposed pigs before or just after inoculation of the resistant strain. All samples concentrated from non-inoculated pigs or before inoculation or their exposure was negative for the *mcr*-1 gene. A total of 19 out of 339 samples proved to be positive and all originated from inoculated pigs, mostly during the first days after inoculation. The absence of differences between contaminated (inoculated) non-treated animals and inoculated and then treated pigs identifies that the colistin treatment, given at the therapeutic dose during the first days of infection, had no detectable impact on pigs regarding the colistin-resistant UB10-260Rif *E. coli* isolates. Probably, colistin given at therapeutic dose does not select these resistant isolates not having a protective effect against an intestinal infection caused by such isolates [105].

#### 3.1.10. Italy

A study in Italy during 2014 and 2015 accomplished to determine the prevalence of colistin resistance and *mcr*-mediated colistin resistance genes and their genetic environment in *E. coli*, extended spectrum beta-lactamase (ESBL)/AmpC-producing *E. coli*, and *Salmonella* spp. in food-animals. All colistin-resistant *E. coli* from pigs tested positive for *mcr* genes, 14 out of 15 harbored *mcr*-1 and one ESBL-producing isolate tested positive for *mcr*-4. Notably, the epidemiology of transferable *mcr*-mediated colistin resistance is evolving rapidly and timely information on prevalence and molecular epidemiology of *mcr*-positive isolates is needed to enhance surveillance and implement measures to prevention and to control further spread of colistin resistance [106].

#### 3.1.11. Belgium

The researchers in Belgium tried to assess the existence of *mcr* genes in an amount of 40 colistin-resistant *E. coli* bacteria isolated from healthy pigs, cattle, and poultry during the years of 2012 to 2016. The study showed that all isolates carried at least one *mcr* gene. The genes observed in this collection were *mcr-1* to *-5*. This study found a triple occurrence (*mcr*-1, *mcr*-3, *mcr*-5); the occurrence of multiple *mcr* genes in a single isolate has been rarely found in other studies and countries [107].

A selection of 105 colistin-resistant *E. coli* strains isolated during 2011 and 2012 from 53 diarrheic piglets from Flanders of Belgium was screened. *Mcr-1* gene was detected in 13 (12,4%) of 105 *E. coli* and seven (13·2%) of 53 were isolated from piglets. These findings proved the importance to identify novel mechanisms and vectors of antibiotic resistance, because of the One Health importance [108].

#### 3.1.12. Brazil

A 2012 research study in Brazil investigated the *mcr-1*-harbouring *E. coli* occurrences in food-producing animals in conjunction with colistin resistance. Thus, 515 isolates originating from food-producing animals were tested. The *mcr*-1 gene was identified in 16 *E. coli* strains exhibiting colistin MICs from 1 to 16 mg/L (MIC50 = 8 mg/L). Two of the *mcr-1* positive *E. coli* strains were collected from faecal samples in 2012 from healthy pigs in farms. The study suggests using colistin only for treatment of clinical infectious diseases and no longer for animal production, so that to diminish the spread of *mcr-1* producing bacteria, achieving the principles of responsible use of antibiotics [109].

#### 3.1.13. Canada

To study occupational exposure to bioaerosols, endotoxins, bacteria, human pathogenic agents (*Staphylococcus aureus*, methicillin-resistant *S. aureus*, *Salmonella* spp., *Mycobacterium avium*, *Clostridium difficile* and *Listeria monocytogenes*), and antibiotic and metal resistance genes (cephalosporin, colistin, zinc), 10 swine confinement buildings (SCBs) were investigated. *Mcr-1* colistin resistance gene was detected in six out of ten SCBs despite restricted use of antibiotics in Eastern Canadian swine herds. This study reinforced the fact that SCBs contain bioaerosols that may contribute to the development of adverse health effects among workers [110].

#### 3.1.14. South Korea

A study in S. Korea investigated the prevalence and antimicrobial susceptibility of *mcr*-harboring colistin-resistant Enterobacteriaceae from food in 2018 and the *mcr*-1 gene was found in *E. coli* isolates from 6.8% (4/59) of pigs. However other *mcr* genes were not detected. The study findings demonstrated that the *mcr-1* gene has emerged and is common in food animals in South Korea [111].

#### 3.1.15. Switzerland

The study focused on the prevalence of intestinal carriage of colistin-resistant and extended-spectrum β-lactamase (ESBL)-producing Enterobacterales. Enterobacterales were collected from pigs in Switzerland in specific farm, where an animal health and antibiotic stewardship program was under progression. Only three colistin-resistant Enterobacterales isolates were detected, from which two of them were *K. pneumoniae* and one *E. cloacae* isolate. Researchers conducted screening in these three isolates, searching for the plasmid *mcr-1* to *mcr-9*, but they did not detect any of them. Obviously, a low prevalence of colistin-resistant Enterobacterales was found in this study [112].

#### 3.1.16. European Studies

A new *mcr-4* colistin resistance gene was identified in Italy in 2013 and then in Spain and Belgium in 2015 and 2016. The relevance of the new *mcr-4* gene was identified for the first time, in different European countries. More specifically, a PCR screening for *mcr* gene was performed in a collection of 125 isolates: 32 strains were positive for *mcr-1*, *mcr-3* for *mcr-2* and 11 for the new *mcr-4* gene. The eleven *mcr-4*-positive strains were also defined positive for the ColE10 replicase and showed a MIC ≥ 4 mg/L for colistin. The *mcr-4* gene was detected in a monophasic variant of *Salmonella* Typhimurium isolated from a pig slaughterhouse in Italy. Nine of 43 *E. coli* Spanish piglets were positive, 37% (9/24) of strains with a colistin MIC > 2 mg/L. Among the Belgian strains, two *E. coli* isolates were *mcr-4* positive among 15 colistin-resistant isolates were found. These findings show that the dissemination of the new *mcr-4* gene in Europe is considerable [40].

One year after, another European study occurred evaluating the presence of the *mcr-1*-like and *mcr-2*-like genes from samples of *E. coli* and *Salmonella* spp. isolated from healthy food-producing animals at slaughter between 2002 and 2014. The *mcr-1*-like gene was detected in 68 *E. coli* (0.7% of all isolates; 45.9% of colistin-resistant isolates) and two *Salmonella* spp. (0.1% of all isolates; 2.2% of colistin-resistant isolates). The 70 *mcr-1*-like-positive isolates collected from animals of Germany, Spain, the Netherlands, and France. Among *E. coli* samples from pigs, the percentage of *mcr-1*-like-positive isolates was low (0.5%) in 2008–2009 but increased to 1.7% in 2013–2014. None of the isolates was found to be positive in the *mcr-2* gene [113].

### 3.2. Cattle

The studies about cattle originated from 12 countries, with Belgium having most of the reviews (3), followed by China (2), France (2) and Portugal, Vietnam, Brazil, Spain, South Korea, Italy, Greece and Netherlands having one study each. There is also one review from various European countries. Most of these manuscripts study *Escherichia coli* isolates (15) while some study Enterobacterales members (1) and *Salmonella* spp. (2). The findings are organized in Table 2.

#### 3.2.1. The Netherlands

To better understand the *mcr-1* gene epidemiology, isolates from livestock and meat were molecularly characterized. *E. coli* strains were isolated from 15 calve faecal samples. The IncHI2 carrying *mcr-1* plasmids were identified in nine *E. coli* isolates derived from calves. The first finding of a chromosomally located *mcr-1* gene in two *E. coli* isolates from veal calves was published [114].

#### 3.2.2. Belgium

The researchers in Belgium tried to assess the existence of *mcr* genes in an amount of 40 colistin-resistant *E. coli* bacteria isolated from healthy pigs, cattle, and poultry during the years of 2012 to 2016. The study showed that all isolates carried at least one *mcr* gene. The genes observed in this collection were *mcr-1* to *-5*. All isolates were MDR and carried between one and nine different replicons. Among 40 *E. coli isolates*, the researchers found 17 different sequences [107].

A total of 105 colistin-resistant *E coli* strains isolated from 52 diarrheic animals in the Wallonia region of Belgium were screened for the presence of *mcr-1. Mcr-1* gene was found in 13 (12.4%) of 105 *E coli* isolates of which 6 (11.5%) of the 52 strains were originated from calves. A marked presence of *mcr-1* in animal pathogenic bacteria in Europe was exhibited [108].

The next study objective in Belgium was the detection of colistin-resistant bovine genes between the years of 2013 and 2018 from less than three months old calves suffering from enteritis or septicemia. Among 158 isolates, 22 were positive using the *mcr-1* PCR and 14 using the *mcr-2* PCR, but *mcr-3*, *mcr-4* or *mcr-5* genes were not detected. Interestingly, only the *mcr-1* and *mcr-2* genes were detected in the 36 PCR-positive bovine *E. coli* isolates, although the other three *mcr* genes have already been identified among bovine. The PCR-negative *E. coli* isolates are possible to carry one of the more recently described *mcr* genes [32,34,46] which had not been detected in bovine *E. coli* to that date [115].

#### 3.2.3. China

In 2016, the colistin-resistant *E. coli* EC11 strain was isolated from cattle feces. WGS was used to outline the mechanism of colistin resistance in this strain. In Bai’s study only the *E. coli* isolate EC11 carried the *mcr-1* gene, and no isolates were detected to carry *mcr-2* or *mcr-3*. This study reported the first record of an *mcr-1* producing *E. coli* EC11 strain belonging to the ST278 lineage. It also proved that *mcr-1* has a great possibility of vertical transmission and may become even more widespread and prevalent in the future [67].

The following year, Zhan and collaborators determined the occurrence of colistin resistance and the epidemiology of *mcr-1* and *mcr-2* mediated colistin resistance genes among cattle. A total of 156 faecal samples were collected from calves, growing cows and milking cows at two farms that had barely used colistin. One of them was located close to a poultry farm while the other at the downwind area of a poultry farm. For cattle, the *mcr-1* PCR product was detected in *E. coli* isolated in 57.14% (4/7) of calves, 75.00% (12/16) of growing cows, and 73.68% (14/19) of milking cows. Similarly, *mcr-2* gene was detected in *E. coli* isolated in 28.57% (2/7) of calves, 12.50% (2/16) of growing cows, and 21.05% (4/19) of milking cows and *E. coli* yielding both *mcr-1* and *mcr-2* geneswas isolated in 6.25% (1/16) of growing cows, and 15.79% (3/19) of milking cows, but not in calves. In conclusion, it was obvious that further dissemination of *mcr* between food-producing animals and humans should be hindered [79].

#### 3.2.4. France

ESBL-positive *E. coli* isolates collected in France were screened so as to determine colistin resistance. Isolates were collected between 2005 and mid-2014 from feces of diarrheic veal calves at farms. Of 517 ESBL-producing *E. coli* isolates collected, 106 (21%) were found to be *mcr-1* positive. Notably, the oldest *mcr-1*-positive *E. coli* isolate from veal calves had been collected in 2005 [116].

As we know, *E. coli* causes diarrhea in calves and, despite the fact that use of drugs from various antibiotic families should be used with caution, determinations of colistin and polymyxin MICs with and without EDTA were performed and the results indicated that eight strains were resistant to colistin and polymyxin B, because of the modification of LPS structure as evidenced by EDTA effect [117].

#### 3.2.5. Portugal

A study conducted in Portugal, included 1206 *E. coli* isolates from various animals. From those, 350 were collected from bovine at slaughter, 12 from bovine meat samples at retail stores, and 20 clinical samples were collected from food-producing animals (stool specimens from sick animals or intestinal contents from deceased animals). Additionally, 634 *Salmonella enterica* isolates were recovered. Overall, *mcr-1*-like genes were detected in 100 colistin-resistant Enterobacteriaceae isolates (97 *E. coli* and 3 *S. enterica*) in various samples and animals. Despite the inclusion of various bovine samples in the study, none of them was detected to carry the *mc-1* gene. Furthermore, none of the isolates was positive for the *mcr-2* gene [47].

#### 3.2.6. Vietnam

A total of 342 ESBL- or AmpC-producing *E. coli* isolates from 330 samples of meat or seafood products (beef, pork, chicken, fish, or shrimp) were isolated during the period of 2012 to 2014 in Ho Chi Minh City in Vietnam. Of the 342 strains, 261 strains were selected and used for the experiments, but no colistin-resistant *E. coli* strain was found [104].

#### 3.2.7. Brazil

Enterobacteriaceae isolates from different sources were screened, to define colistin resistance and *mcr-1* gene presence in Brazil during 2000–2016. From 158 bovine, 22 samples were isolated, but no colistin-resistant Enterobacteriaceae isolate was found to be positive in *mcr-1* harboring genes [109].

#### 3.2.8. Spain

The aim of a study was to investigate the possible existence of *mcr-1* and *mcr-3* genes in *E. coli* isolates derived from cattle. An *E. coli* isolate carrying the *mcr-3* gene was detected among other isolates expressing colistin resistance. It was sampled in cattle faeces at slaughter in Spain in September 2015. A total of six *E. coli* isolates were resistant to colistin and for that reason were further characterized and three genotypes were identified: strains carrying only *mcr-1*, a strain carrying *mcr-1* and *mcr-3.2*, and strains without any plasmids [53].

#### 3.2.9. S. Korea

In a Korean study, 150 fecal samples from healthy food animals were isolated. Among them, 57 were from cattle across the country and farmers’ markets. No colistin-resistant *E. coli* strain was recovered from cattle [111].

#### 3.2.10. Italy

The aim of an Italian study was to provide information on the epidemiology of colistin-resistant recovering *mcr*-positive *Escherichia coli* and *Salmonella* isolates in bovine, less than one year old, during the period of 2014 and 2015. Most colistin-resistant isolates harbored *mcr-1* genes, but four *mcr-3* genes were detected in ESBL-producing *E. coli* isolates from bovines, and two *mcr-4*, one in an ESBL and one in an indicator *E. coli* isolate from pigs and bovines. Additionally, *mcr-3.2* and *mcr-4.3* genes in *E. coli* from bovines were described. These findings clarify the epidemiology of colistin resistance in food-producing animals in Italy along with its genetic background, highlighting the possibility of horizontal transferring of *mcr* from commensal bacteria to major food-borne pathogens [106].

#### 3.2.11. Greece

A survey analyzing milk samples collected from cows with mastitis was performed in order to search for the occurrence of extended-spectrum β-lactamase (ESBL)–producing *E. coli*. A total of 89 *E. coli* isolates out of 400 milk samples (22.25%) were collected from 12/23 (52%) farms. Six isolates originating from six cows on a single farm were ESBL producers, *mcr-1*-positive and resistant to colistin. This was the first report of endemic mastitis caused by *mcr-1*-positive, ESBL-producing *E. coli* in the country [118].

#### 3.2.12. Europe

In the El-Garch et al. study, the presence of the *mcr*-1-like and *mcr*-2-like genes was investigated in a collection of *E. coli* and *Salmonella* spp. isolated from bovines at slaughter during 2002 and 2014, in Europe. No isolates from cattle were found positive in harboring *mcr*-1 and *mcr-2* genes [113].

### 3.3. Poultry

Colistin resistance studies in chicken originated from 18 countries. China is the country having the most studies, six in the number, followed by Bangladesh (4), Netherlands (2), Lebanon (2), Vietnam (2), Brazil (2) and then Portugal, Iraq, South Korea, Europe, Romania, Paraguay, Tunisia, Algeria, Pakistan, Turkey, Belgium, Nigeria with one review. In addition, there is one review that occurred in different countries of Europe. A total of 25 of the studies isolate *E. coli* bacteria, three reviews investigated various Enterobacteria; *Salmonella* spp. was investigated in five reviews and *A. baumannii* in one review. The findings are organized in Table 3.

#### 3.3.1. China

A study investigated the prevalence of colistin-resistant avian-pathogenic *E. coli* (APEC) and the possibility of transmission of *mcr*-1 gene to APEC. Between March 2017 and December 2018 in Anhui Province in China, a total of 72 APEC isolates were collected. After that, the isolates were screened for the *mcr*-1 gene. Three APEC isolates (AH25, AH62, and AH65) were carrying the *mcr*-1 gene and expressed multidrug resistance. The *mcr*-1 genes were located on IncI2 plasmids and these plasmids were transferrable. Particularly, despite strains AH62 and AH65 both belonged to ST1788 and were collected from different places, they shared very similar plasmids with the same drug resistance genes. This study brings to light the possibility of an epidemic of *mcr*-1-positive APEC. For this reason, monitoring is highly recommended [119].

Wang and collaborates discussed the possibility of increased antibiotic resistance transmission due to live poultry trade. When colistin use was banned as a growth promoter the prevalence of *mcr-1* decreased, but feces from poultry farms are a reservoir of antimicrobial resistant bacteria and antibiotic resistance genes (ARGs). There is an important interface between humans, farm animals, and environments, and live poultry markets considered to be a reservoir for ARGs and viruses. However, the way of transportation from this interface remains unknown. The mobile ARGs identified in chickens and the distribution of the *mcr*-family genes was analyzed. In addition, the study explored the prevalence of *mcr*-1 in live poultry markets after banning colistin-positive additives in China. Mcr-1 prevalence decreased significantly in these markets across seven provinces in China, from 190/333 (57.1%) samples in September 2016–March 2017 to 208/544 (38.2%) samples in August 2018–May 2019. It is also remarkable that *mcr*-10 was identified in chicken gut microbiomes (1.5%, 14/910) [120].

Colistin resistance gene *mcr-1* was detected in four *Salmonella* serovars isolated from humans and diarrheic animals, during 2010 and 2015 in Taiwan. The resistance gene was carried on different plasmids. One *mcr-1*-carrying conjugative plasmid, a variant of pHNSHP45, was disseminated among *Salmonella* isolates recovered from humans, pigs, and chickens. For that reason, *mcr-1* is widespread and has become prevalent in zoonotic pathogens in this country allowing us to speak about the importance of One Health [70].

In this study, the PCR method was used to detect *mcr* genes (*mcr-1*, *mcr-2*, *mcr-3*). In total, 1696 cloacal and 1647 oropharyngeal samples were collected from live poultry from markets in 24 provinces. The *mcr-1* prevalence in chickens was 31.8%, the *mcr-2* prevalence was 5.5% and the *mcr-3* prevalence was 5.2%. The study identified 33 new *mcr-2* variants and 12 new *mcr-3* variants. This study indicated the high prevalence of *mcr* genes in Chinese poultry [73].

The presence of the recently identified colistin resistance genes *mcr-4*, *mcr-5*, in China was determined with the PCR method. In total, 1.647 oropharyngeal samples collected from poultry at live-bird markets, in 24 provinces of China. The *mcr-4* gene was found in chickens in 17.2% and the *mcr-5* was identified in 9.9% of the samples. This study further proved the presence of the *mcr-4* and *mcr-5* in chickens in China [76].

A year later, a similar study tried to investigate the colistin resistance and *mcr-1* and *mcr-2* genes spread in domestic animals in Jiangsu Province. Fecal swabs from chickens at different ages were collected. The prevalence of *mcr-1* in colistin resistant *E. coli* isolates was 87.58% (388/443), *mcr-2* was 14.90% (66/443). Co-occurrence of *mcr-1* and *mcr-2* was also identified in 7.22% (32/443) of chickens and proved that the *mcr* dependent mechanism dominated in *E. coli* [79].

#### 3.3.2. Belgium

The researchers in Belgium tried to assess the existence of *mcr* genes in an amount of 40 colistin-resistant *E. coli* bacteria isolated from healthy pigs, cattle, and poultry during the years of 2012 to 2016. The study showed that all isolates carried at least one *mcr* gene. Only one *mcr-1* gene was detected in a broiler chicken sample [107].

#### 3.3.3. The Netherlands

To obtain a better understanding of the epidemiology of the *mcr-1*-positive isolates, samples collected from livestock and meat were molecularly characterized. In total, 10 *E. coli* samples were collected from broiler feces. In addition, 13 *Salmonella* samples were obtained from broiler meat and imported turkey meat. All isolates exhibited reduced susceptibility to colistin. Specifically, in five out of ten samples, *E. coli* isolates were found to be *mcr-1* carriers in chickens, as was in all of the *Salmonella* samples. The frequent finding of *mcr-1*-positive IncHI2/ST4 plasmids in *E. coli* isolates in this study indicates the importance of this plasmid’s role in the spread of *mcr-1* gene in livestock, as also proved in previous studies [114].

The presence of *mcr-1* and *mcr-2* in Dutch retail chicken meat was also studied in 2017. The occurrence of *mcr-1* was 24,8%, whereas *mcr-2* gene was not detected at all. The presence of *mcr-1*-positive Enterobacteriaceae was 64,2%. Every supermarket chain had a different prevalence and was lower in free-range chicken samples. The high prevalence of the *mcr-1* gene in food should be taken under consideration [121].

#### 3.3.4. Brazil

The *mcr-1* gene was detected in 14 *E. coli* strains originated from faecal samples of healthy chickens. The samples had been gathered in 2013 from farms located in Paraná, São Paulo, and Minas Gerais states. All 14 isolates had a MIC ≥ 8 mg/L. Brazil is the third largest chicken meat producer, after the United States and China, and the largest exporter of this product (U.S.I.T.C., 2012). This is very important considering the contamination of the *mcr-1* gene worldwide [109].

As colistin has been widely used in animal feed as a growth promoter in Brazilian livestock, mainly in pigs and poultry, this study researched eight colistin-resistant *E. coli* isolates carrying *mcr-1* isolated from commercial chicken meat in west regions of São Paulo in Brazil. However, plasmid characterization by PCR-based replicon typing revealed the presence of IncX4-type plasmids in five *mcr-1*-positive *E. coli* isolates. After this confirmation the use of colistin in animal feed was prohibited by MAPA (Regulatory Instruction No. 45]) in November 2016, following the international recommendations of the World Health Organization [122].

#### 3.3.5. Vietnam

Twelve chicken farms were assessed in Vietnam measuring antimicrobial usage and investigating the prevalence of antimicrobial resistance and their corresponding molecular mechanisms among 180 *Escherichia coli* isolates. The *mcr-1* gene was present in 19 out of 180 samples that were in agreement with phenotypic colistin resistance [101].

Another study investigated local foods in Vietnam for contamination with colistin-resistant bacteria. A total of 342 ESBL- or AmpC-producing *E. coli* isolates from 330 samples of meat or seafood products (beef, pork, chicken, fish, or shrimp) were collected from 2012 to 2014 in Ho Chi Minh City in Vietnam. The presence of the *mcr-1* gene in chicken *E. coli* isolates was 39% (56/144). No other *mcr* genes were detected in chickens [104].

#### 3.3.6. Portugal

A study investigated the presence of *mcr-1 and mcr-2* genes in Enterobacteriaceae isolates from food-producing animals, meat and its products and animal feed during the years of 2010 and 2015. This study was the most wide-ranging study and the only one, until then, conducted in Portugal, reporting the occurrence of *mcr*-1 genes in *E. coli* isolates from broilers (2%, 4/202). No *mcr-2* gene was detected. It was also found that fattening broilers were negatively associated with colistin resistance [47].

#### 3.3.7. Romania

This study evaluated the occurrence of *mcr-1* and *mcr-2* plasmid and/or carbapenem resistance in human clinical Enterobacterales and other Gram-negative bacteria (*n* = 543) as well as third generation cephalosporin resistance. Totally, 11 *mcr-1* positive *E. coli* isolates were collected, and the *mcr-2* gene was not detected in any of the poultry isolates. In addition, *mcr-1* and *mcr-2* genes were not found in any fecal isolates from workers and none of the 543 colistin and/or carbapenem resistant human clinical isolates were proved to be positive. A high prevalence was reported; a total of 11.9% *mcr-1* plasmid mediated colistin resistance was detected in commensal 3GCR AmpC producing *E. coli* from poultry sampled in 2011/2012 reported [123].

#### 3.3.8. Bangladesh

As colistin has also been used in Bangladesh poultry, a study was conducted so that to present prevalence and molecular basis of colistin-resistance of *E. coli* (CREC) in breeder poultry of 108 farms. The study was carried out in a period of one year and demonstrated the detection of 24 (26.1%) CREC isolates among which a total of 62.5% carried one or more *mcr* gene(s) as confirmed by PCR. The result of the study was that the researchers detected eight *mcr*-1, one *mcr*-2 and four *mcr*-3 genes using PCR [124].

Another study in Bangladesh by Islam and his colleagues investigated droppings of poultry chickens and household native chickens. In this study, researchers recorded the phenotypic colistin-resistance and the prevalence of colistin-resistance *mcr-1* gene in bacteria from their samples. Overall, using disk-diffusion assessment method, the study detected 39.6% (59/159) isolates showing colistin-resistance. The resistance prevalence in poultry-chicken isolates was higher (48.5%, 48/99) than in native-chicken isolates (22%, 11/50; *p* = 002). With the use of colistin, the appearance of *mcr-1* was associated with phenotypic colistin-resistance phenomena in a higher portion (*p* = 0.06) than without the use of it (*p* < 0.001) [125].

In 2021, Uddin and colleagues conducted a study in Bangladesh, in which they investigated the colistin resistance gene *mcr*-1. Furthermore, the researchers determined its in-silico functional analysis in *Salmonella* isolates. Out of 100 samples from chicken liver and intestine, 82 *Salmonella* spp. were isolated and characterized. All isolates presented high resistance to colistin, a percentage of 92.68%. In addition, 10 *Salmonella* isolates randomly selected were analyzed by PCR searching for colistin resistance *mcr -1* genes. Five of them were positive to the presence of the *mcr-1* gene originated from *Salmonella* spp. [126].

Two years before the previous study, in 2019, a study was carried out in the country so that to detect the antibiotic resistance and genes in *E. coli*. In two different live bird markets of Chattogram, sixty cloacal swab samples were collected from healthy broilers. The researchers targeted the presence of *E. coli* in broilers; Isolates were detected in 61.67%, and out of this a total of 48.65% were found to be sensitive to colistin. For that reason, researchers concluded that the presence of *mcr* genes in *E. coli* isolates in broilers could be a danger for humans, animals, and their environment [127].

#### 3.3.9. Iraq

*Acinetobacter baumannii* is a bacterium with antibiotic resistance. For that reason, *A. baumannii* strains from animals were isolated. The aim was to identify *A. baumannii* antibiotic resistance and infectious features from chicken raw meat. Colistin resistance was detected among 12% of the isolates and the existence of *mcr-1* genes was in 13% of them. Hence, early detection combined with determination of resistance profile and rigorous control strategies are very important for One Health and spreading prevention [128].

#### 3.3.10. South Korea

This study was performed to investigate the occurrence and antimicrobial resistance of *mcr*-harboring colistin-resistant Enterobacteriaceae from food animals in South Korea in 2018. In this study 34 chicken samples from healthy animals were obtained. The *mcr-1* gene was detected in *E. coli* in two of the thirty-four isolates (5.9%). In addition, no other *mcr* genes were detected. Both of the *mcr-1*-positive chicken isolates of the study in general showed multidrug resistance and co-produced β-lactamases [111].

#### 3.3.11. Tunisia

A Tunisian study aimed to genetically characterize multidrug-resistant *E. coli* isolates from broiler chickens that died from colibacillosis in three farms from Tunisia. A high infection rate of *E. coli* was observed (50%). Most of the *E. coli* isolates were multidrug-resistant (96%) and among them 24% were colistin-resistant. Seven out of 12 colistin-resistant isolates carried the *mcr-1* gene. This is the first report of ESBL-*mcr-1* carrying *E. coli* isolates from chickens suffering from colibacillosis in Tunisian poultry farms [129].

#### 3.3.12. Pakistan

For the purposes of a study in Lahore city in Pakistan, 150 cloacal swabs were collected from broilers in retail shops, so that to be processed for isolation of *E. coli*. A total of 100 *E. coli* isolates were collected and were tested for antimicrobial sensitivity to colistin sulfate using broth dilution method. After that, the isolates that showed colistin resistance were tested by PCR for *mcr*-1 and *mcr*-2 genes of colistin resistance. Out of 100 isolates, 59 showed resistance to colistin, while resistance existed in many other antibiotics tested as well. Among those 59 isolates, only seven isolates were carrying the *mcr-1* gene, but the *mcr-2* gene was not detected. The conclusion of the study is that the presence of *mcr*-1 gene in *E. coli* in commercial birds can be a reason of spreading in humans and environment [130].

#### 3.3.13. Nigeria

As in Nigeria the poultry industry is a main livestock sector, a study occurred in the southeast part of the country, in order to screen colistin-resistant Enterobacterales from poultry birds and determine the genetic similarity of *mcr*-harboring isolates. Between March and November 2018, the amount of 785 faecal and cloacal swab samples were collected from chickens in 17 farms located in three neighboring states. Out of 785 samples, 45 were positive for colistin-resistant Enterobacterales, and among them, 23 carried the *mcr*-1 gene (22 *E. coli*, 1 *K. pneumoniae*). Furthermore, two *E. coli* isolates found harboring a new allelic variant, the *mcr*-1.22 [131].

#### 3.3.14. Turkey

The Turkish study aimed to investigate the colistin resistance in *E. coli* and the presence of the *mcr*-1 gene. For that reason, a total number of 200 *E. coli* isolates were collected from broilers’ faeces and intestinal samples. The *mcr*-1 gene was not detected in any of the samples, notwithstanding some isolates exhibited phenotypic colistin resistance. For that reason, the study concluded that the reason of colistin resistance, despite of the lack of *mcr*-1 gene, was potentially of chromosome origin or that there were other genes responsible for resistance [132].

#### 3.3.15. Paraguay

A total of 62 *E. coli* and 22 *K. pneumoniae* isolates were collected coming from 12 farms in Paraguay. Among them, representative isolates were subjected to WGS. Resistance to colistin was observed in 29 (out of 62) of *E. coli* isolates and WGS revealed these colistin-resistant isolates carried the *mcr-5.1* gene. None of the *K. pneumoniae* isolates carried *mcr-5.1*. This Paraguayan study indicates for one more time that poultry farms are the reservoir of antibiotic resistance [133].

#### 3.3.16. Lebanon

The high prevalence of multidrug-resistant (MDR) *E. coli* carrying *mcr*-1 was also reported in poultry of Lebanon. For that reason, the study of 2021 performed WGS in fecal samples of poultry and detect genomic similarities in five of the isolates. The results of the WGS analysis are that the strains showed resistance in nine antibiotics, out of 19, because of gene harboring, including *mcr-1.1* gene and other genes responsible for resistance in important antibiotics used in agriculture and human medicine. Furthermore, the strains associated with zoonotic transmission from poultry to humans, food contamination and clinical samples belonged to different STs [134].

Another study of the country, during the same year indicates the prevalence of *mcr*-1-positive *E. coli* in poultry. The samples were collected from 32 farms among three Lebanese governorates. The poultry of these farms were slaughtered in the same place. The result of the study is that out of 32 farms, 27 (84.4%) were positive to the presence of the *mcr*-1 gene. A total of 84 *E. coli* samples were collected, of which 62 harboring the *mcr*-1 gene. In addition, other numerous resistances were identified. It was also noted that the *mcr*-1 gene was carried by 36 IncX4 and 24 IncI2 plasmids. These plasmids are both known for their efficient transfer capacities [135].

#### 3.3.17. Algeria

Chaalal and his colleagues in Western Algeria aimed to investigate the importance and genetic characteristics of colistin-resistant studying Enterobacterales isolated from chicken meat. They visited three farms, in different provinces in Western Algeria and they collected 181 samples of chicken meat. A total of 22 isolates indicated colistin resistance with the prevalence of 12.2% (22/181). Among them, 17 isolates were *E. coli* and five were *K. pneumoniae*. Specifically, the *mcr-1* gene was detected in 11 *E. coli* isolates (6.1%, 11/181) and was associated with IncFV (*n* = 7) and IncFIIK (*n* = 4) plasmids [136].

#### 3.3.18. Europe

A European study in 11 countries detected the presence of the *mcr-1*-like and *mcr-2* genes in *E. coli* and *Salmonella* spp. isolated from healthy food-producing animals at slaughter during the years of 2002 and 2014. Among the 10,206 *E. coli* and 1774 *Salmonella spp*. samples originated from cattle, pigs, and chickens, 148 *E. coli* and 92 *Salmonella spp*. isolates found to be resistant to colistin. *Mcr-1*-like-positive *E. coli* was found in the 44.4% of chicken samples isolated from 2008 to 2014, whereas none of the isolates found positive for *mcr-2*. From *Salmonella* isolates, respectively, only one, originating from Germany, was *mcr-1*-like-positive and was isolated from a chicken. The results showed that *mcr-1*-like gene had a low prevalence in food-producing animals at slaughter in European countries, but the high diversity of *E. coli* made the horizontal transfer of *mcr-1* possible [113].

**Table 1:** Swine.

	Country	Bacteria	*mcr* Genes Were Searched	Number of Isolated Samples	Colistin Resistant Samples	*mcr* Number	*mcr*	% *mcr* Prevalence	Year	Citation
1	Japan	*E. coli*	*mcr-1 to mcr-8*	90 pre-ban, 511 after-ban	23 pre-ban, 19 after-ban	23 pre-ban, 19 after-ban	*mcr-1*	25% pre-ban, 4% after-ban	2021	[94]
2	Belgium	*E. coli*	*mcr-1*, *mcr-2*, *mcr-3*, *mcr-4*, *mcr-5*, *mcr-6*, *mcr-7*, *mcr-8*, *mcr-9*, *mcr-10*	40	40	3	*mcr*-1	9.6	2021	[107]
3	Switzerland	*E. coli*	*mcr-1*, *mcr-2*, *mcr-3*, *mcr-4*, *mcr-5*, *mcr-6*, *mcr-7*, *mcr-8*, *mcr-9*		81	3	mcr-1 to mcr-9	0	2021	[112]
4	Thailand	*Salmonella*	*mcr-1*, *mcr-2*, *mcr-3*	300	4	5	*mcr*-3	1.3	2021	[83]
5	Thailand, Cambodia, Lao, Myanmor	*E. coli* *Salmonella*	*mcr-1*, *mcr-2*, *mcr-3*, *mcr-4*, *mcr-5*, *mcr-6*, *mcr-7*, *mcr-8*, *mcr-9*, *mcr-10*	809	80	68	*mcr*-1	62.5	2021	[82]
						31	*Mcr*-2	6.25		
6	Spain	*E. coli*	*mcr-1*, *mcr-2*, *mcr-3*, *mcr-4*, *mcr-5*	70	15	14	*mcr*-1	20	2020	[90]
						1	*mcr*-4	1.42		
7	China	*E. coli*		115	10	10	*mcr*-1	8.70	2019	[78]
8	China	*E. coli*, *Klebsiella pneumoniae*, *Kluyvera ascorbata*, *Enterobacter cloacae*	*mcr-1*, *mcr-2*, *mcr-3*, *mcr-4*, *mcr-5*, *mcr-6*, *mcr-7*, *mcr-8*	65	33	28	*mcr*-1	43.07	2019	[80]
						4	*mcr*-1 +*mcr*-3	6.15		
						1	*mcr*-3	1.54		
9	China	*E. coli*	*mcr-1*, *mcr-2*	811	440	303	*mcr*-1	37.36	2019	[79]
						88	*mcr*-1+ *mcr*-2	10.85		
						206	*mcr*-2	25.40		
10	China	*E. coli*	*mcr-1*	600	457	152	*mcr*-1	25.33	2018	[72]
11	China	Enterobacteriaceae, *Aeromonas hydrophila*, *Ε. Coli*, *A. Veronii*, *A. Caviae*	*mcr-1*, *mcr-2*, *mcr-3*, *mcr-4*, *mcr-5*	336	8	1	*mcr*-5	0.30	2018	[77]
12	China	*E. coli*	*mcr-4*, *mcr-5*	1552	1454	621	*mcr*-4	40.01	2018	[76]
						478	*mcr*-5	30.80		
						266	*mcr*-*4 +mcr-5*	17.14		
13	China	Enterobacteriaceae, *Moraxella* spp., *Aeromonas Veronii*	*mcr-1*, *mcr-2*, *mcr-3*	4895	1454	1152	*mcr*-1	23.53	2018	[73]
						818	*mcr*-2	16.71		
						272	*mcr*-3	5.55		
14	China	*E. coli*	*mcr-1*, *mcr-2*, *mcr-3*, *mcr-4*, *mcr-5*	417		71	*mcr*-1	17.02	2018	[75]
						5	*mcr*-3	1.20		
15	China	*E. coli*	*mcr-1*	204	81	78	*mcr*-1	38.23	2018	[71]
16	Japan	*E. coli*	*mcr-1*, *mcr-2*, *mcr-3*, *mcr-4*, *mcr-5*	676	120	36	*mcr*-1	5.32	2018	[96]
						10	*mcr*-3	1.15		
						34	*mcr*-5	5.02		
17	Great Britain	*Moraxella spp*., *E. coli*	*mcr-1*, *mcr-2*	657		0	*mcr*-1	0	2017	[32]
18	Italy, Spain, Belgium	*Salmonella enterica serovar*, *Typhimurium*, *Salmonella enterica*, *E. coli*	*mcr-1*, *mcr-2 mcr-3*	125	50	32	*mcr*-1	25.6	2017	[40]
						3	*mcr*-2	2.40		
						11	*mcr*-4	8.80		
19	Germany	*E. coli*	*mcr-1*	216	26	12	*mcr*-1	5.55	2018	[93]
20	China	*E. coli*	*mcr-1*	1026	302	302	*mcr*-1	30	2016	[69]
21	Germany	Enterobacteriaceae	*mcr-1*, *mcr-2*	436	43	15	*mcr*-1	3.44	2017	[92]
22	China	*Salmonella spp*.	*mcr-1*	279	20	7	*mcr*-1	2.50	2017	[70]
23	Great Britain	*E. coli*	*mcr-1*	556	163	8	*mcr*-1	1.44	2017	[97]
24	China	*E. coli*	*mcr-1*	93	10	2	*mcr*-1	2.15	2016	[67]
25	China	*E. coli*	*mcr-1*		16	6	*mcr*-1	37.5	2016	[68]
26	Vietnam	*E. coli*	*mcr-2*	250	180	17	*mcr*-1	6.80	2016	[101]
27	Belgium	*E. coli*	*mcr-1*	105	52	7	*mcr*-1	6.67	2016	[108]
28	Germany	*E. coli*	*mcr-1*	557	4	3	3*mcr*-1		2016	[91]
29	South Korea	*E. coli*	*mcr-1*, *mcr-2*, *mcr-3*, *mcr4*, *mcr-5*, *mcr-6*, *mcr-7*, *mcr-8*	59	4	4	*mcr*-1	6.78	2020	[111]
30	Canada	*Salmonella spp*. *E. coli*	*mcr-1*	10	6	1	*mcr*-1	0.10	2019	[110]
31	Portugal	*E. coli*, *Salmonella*	*mcr-1*	398	42	40	*mcr*-1	10.05	2019	[47]
32	Spain	*Citrobacter freundii*, *E. coli*, *Klebsiella pneumoniae*, *Elisabethkingia* spp.	*mcr-1*, *mcr-2*, *mcr-3*, *mcr-4*	76	72	63	*mcr*-1	82.90	2019	[87]
						3	*mcr*-4	3.95		
33	France	*E. coli*	*mcr-1*	339	86	19	*mcr*-1	5.60	2019	[105]
34	Italy	*E. coli*, *Salmonella*	*mcr-1*, *mcr2*, *mcr-3*, *mcr-4*, *mcr-5*	304	14	13	*mcr*-1	0.33	2018	[106]
35	Vietnam	*E. coli*	*mcr-1*, *mcr-2*, *mcr-3*, *mcr-4*, *mcr-5*	261	62	60	*mcr*-1	97	2018	[104]
						2	*mcr*-3	0.77		
36	China	*E. coli*	*mcr-1*	15193	1416	274	*mcr*-1	1.80	2020	[81]
37	Japan	*E. coli*	*mcr-1*	684	309	90	*mcr*-1	13.16	2016	[95]
38	Brazil	Enterobacteriaceae	*mcr-1*	113	79	2	*mcr*-1	1.77	2016	[109]
39	Europe	*E. coli*, *Salmonella spp*.	*mcr-1*, *mcr-2*	3510	78	25	*mcr*-1	0.81	2018	[113]
40	China	*E. coli*	*mcr-3*	6497	49	4	*mcr*-3	0.06	2018	[74]
41	Portugal	*E. coli* *Klebsiella pneumoniae* *Enterobacter Aerogenes* Enterobacteriaceae	*mcr-1*	93	62	12	*mcr*-1	12.90	2020	[100]
42	Spain	*E. coli*	*mcr-1*, *mcr-2*, *mcr-3*, *mcr-4*, *mcr-5*	200	43	14	*mcr*-1	7	2020	[88]
						26	*mcr*-4	13		
						6	*mcr*-5	3		

**Table 2:** Cattle.

	Country	Bacteria	*mcr* Genes Were Searched	Number of Isolated Samples	Colistin Resistant Samples	*mcr* Number	mcr	% mcr Prevalence	Year	Authors
1	Belgium	*E. coli*	*mcr-1*, *mcr-2*, *mcr-3*, *mcr-4*, *mcr-5*, *mcr-6*, *mcr-7*, *mcr-8*, *mcr-9*, *mcr-10*	40	40	27	*mcr-1*	87.1	2021	[107]
2	China	*E. coli*	*mcr-1*, *mcr-2*	156	42	30	*mcr-1*	71.43	2019	[79]
						8	*mcr-2*	19.05		
						4	*mcr-1 + mcr-2*	9.52		
3	Europe	*E. coli*, *Salmonella spp*.	*mcr-1*, *mcr-2*	2553	32	0	*mcr-1*	0	2018	[113]
4	Belgium	*E. coli*	*mcr-1*	105	52	13	*mcr-1*	12.38	2016	[108]
5	S. Korea	*E. coli*	*mcr-1*, *mcr-2*, *mcr-3*, *mcr-4*, *mcr-5*, *mcr-6*, *mcr-7*, *mcr-8*, *mcr-9*	57	0	mcr-1	*mcr-1*	0	2020	[111]
6	Greece	*E. coli*		400	89	6	*mcr-1*	1.50	2020	[118]
7	France	*E. coli*	*mcr-1*	14	9	8	*mcr-1*	57.14	2019	[117]
8	Portugal	*Salmonella*, *E. coli*	*mcr-1*	350	0	0		0	2019	[47]
9	Belgium	*E. coli*	*mcr-1*, *mcr-2*, *mcr-3*, *mcr-4*, *mcr-5*	94	45	9	*mcr-1*	9.57	2019	[115]
10	China	*E. coli*	*mcr-1*, *mcr-2*, *mcr-3*	120		7	*mcr-2*	7.45	2018	[67]
						1	*mcr-1*	0.83		
11	Italy	*E. coli*	*mcr-1*, *mcr-2*, *mcr-3*, *mcr-4*, *mcr-5*	678	8	5	*mcr-1*	2.24	2018	[106]
12	Vietnam	*E. coli*	*mcr-1*, *mcr-2*, *mcr-3*, *mcr-4*, *mcr-5*	29	0	1	*mcr-4*	0.45	2018	[104]
						0	*mcr-1*	0		
13	Netherlands	*E. coli*		15	15	11	*mcr-1*	73.33	2016	[114]
14	France	*E. coli*	*mcr-1*	517	106	75	*mcr-1*	14.50	2016	[116]
15	Brazil	Enterobacteriaceae	*mcr-1*	158	22	0	*mcr-1*	0	2016	[109]
16	Spain	*E. coli*	*mcr-1*, *mcr-2*, *mcr-3*	152	6	5 1	*mcr-1* *mcr-3*	3.29 0.66	2017	[53]

**Table 3:** Chicken.

	Country	Bacteria	*mcr* Genes Were Searched	Number of Isolates	Colistin Resistant Isolates	*mcr* Number	mcr	% mcr Prevalence	Year	Authors
1	Bangladesh	*E. coli*	*mcr-1*	40	24	8	*mcr-1*	33.3	2021	[124]
						1	*mcr-2*	4.16		
						4	*mcr-3*	16.6		
2	Bangladesh	*E. coli*	*mcr-1*	159	59	2	*mcr-1*	34	2021	[125]
3	Algeria	*E. coli* *Klebsiella*	*mcr-1*	181	17	11	*mcr-1*	64.7	2021	[136]
		*pneumoniae*	*mcr-1*		5	0				
4	Bangladesh	*Salmonella*	*mcr-1*	82	10	5	*mcr-1*	50	2021	[126]
5	Pakistan	*E*. *coli*	*mcr-1*	100	59	5	*mcr-1*	15	2021	[130]
6	Libanon	*E. coli*	*mcr-1*	84	32	27	*mcr-1*	84.3	2021	[135]
7	Libanon	*E. coli*	*mcr-1*	93	19	9	*mcr-1*	47.4	2021	[134]
8	China	*Enterobacteriae*	*mcr-1*, *mcr-2*, *mcr-3*, *mcr-4*, *mcr-5*, *mcr-6*, *mcr-7*, *mcr-8*, *mcr-9*, *mcr-10*	910 (gut microbiomes)		293	*mcr-1*	32.2	2021	[120]
						14	*mcr-10*	1.5		
9	China	*E. coli*	*mcr-1*	72	3	3	*mcr-1*	100	2021	[119]
10	Paraguay	*E. coli*	*mcr-5*	62	29	6	*mcr-1*	20.6	2021	[133]
11	Belgium	*E. coli*	*mcr-1 to mcr-10*	40	40	1	*mcr-1*	100	2021	[107]
12	Turkey	*E. coli*	*mcr-1*	200	15	0	*mcr-1*	0	2021	[132]
13	Nigeria	*E. coli*	*mcr-1*, *mcr-2*, *mcr-3*, *mcr-4*, *mcr-5*, *mcr-6*, *mcr-7*, *mcr-8*, *mcr-9*, *mcr-10*	785	45	23	*mcr-1*	62.5	2021	[131]
							*mcr-1.22*	6.25		
14	China	*E. coli*	*mcr-1*, *mcr-2*	1232	443	388	*mcr-1*	31.50	2019	[79]
						66	*mcr-2*	5.36		
						32	*mcr-1 +mcr-2*	2.60		
15	Netherlands	*E. coli*, *Salmonella*	*mcr-1*	10	5	2	*mcr-1*	20	2016	[114]
16	Brazil	Enterobacteriaceae	*mcr-1*	280	113	14	*mcr-1*	12.40	2016	[109]
17	Netherlands	*E. coli*	*mcr-1-mcr2*	214	53	34	*mcr-1*	15.89	2017	[121]
18	Brazil	*E. coli*	*mcr-1*	41	8	5	*mcr-1*	12.19	2017	[122]
19	China	*E. colli*	*mcr-4*, *mcr5*	1836	1498	257	*mcr-4*	14	2018	[76]
20	China	*E. coli*	*mcr-1*, *mcr-2*, *mcr-3*	1836	1498	477	*mcr-1*	25.98	2018	[73]
						82	*mcr-2*	4.47		
						78	*mcr-3*	4.24		
21	Europe	*E. coli*, *Salmonella* spp.	*mcr-1*, *mcr-2*	2973	114	40	*mcr-1*	1.80	2018	[113]
22	China	*E. coli*, *Salmonella spp*.	*mcr-1*, *mcr-3*	450	17	2	*mcr-1*	0.44	2017	[70]
23	Vietnam	*E. coli*	*mcr-2*	180	20	1	*mcr-1*	7.78	2016	[101]
24	Iraq	*A. Baumannii*	*mcr-1*	424	80	2	*mcr-1*	0.47	2020	[128]
25	S. Korea	Enterobacteriaceae	*mcr-1*, *mcr-2*, *mcr-3*, *mcr-4*, *mcr-5*, *mcr-6*, *mcr-7*, *mcr-8*	34	2	2	*mcr-1*	5.88	2020	[111]
26	Romania	*E. coli*	*mcr-1*, *mcr-2*	92	11	17	*mcr-1*	18.47	2019	[123]
27	Bangladesh	*E. coli*	*mcr-1*	60	37	18	*mcr-1*	0.30	2019	[127]
28	Portugal	*E. coli*, *Salmonella spp*.	*mcr-1*	202	6	4	*mcr-1*	1.98	2019	[47]
29	Vietnam	*E. coli*	*mcr-1*, *mcr-2*, *mcr-3*, *mcr-4*, *mcr-5*	144	143	56	*mcr-1*	39.16	2018	[104]
30	Tunisia	*E. coli*	*mcr-1*	50	12	7	*mcr-1*	0.14	2020	[129]

## 4. Discussion

The objective of this study was to present the results of relevant studies on an international level, on the subject of colistin resistance due to *mcr* genes prevalence.

Colistin is widely used in veterinary medicine. In Europe, polymyxins was the fifth most common group of antibiotics, although significant differences between countries and the affected species were observed (available data in http://www.ema.europa.eu/ema, accessed on 27 September 2021).

Nowadays, polymyxins are becoming a last resort antimicrobial in human medicine and for that reason National Competent Authorities and the farming industry are now taking measures to diminish the use of colistin in food-producing animals and especially in poultry and swine, as E.M.A. demanding (EMA/CVMP/CHMP, 2016). The detection of colistin resistance gene in food indicates the importance of One Health as this resistance is a potential public health threat, because of horizontal transmission [31].

A great number of studies shows that meat-producing animals such as swine, bovine and poultry face a great danger by colistin exposition, and they are prone to spread the plasmid-mediated *mcr* gene.

Since the *mcr*-1 gene is the major factor of bacterial colistin resistance, most studies have focused more on this particular one rather than the rest of the *mcr* gene family. After literature search and using PRISMA guidelines principles, a total of 40 swine, 16 bovine and 31 poultry studies were collected concerning *mcr*-1 gene five swine, three bovine and three poultry studies referred to *mcr*-2 gene; eight swine, one bovine, two poultry studies were about *mcr*-3 gene; six swine, one bovine and one poultry manuscript studied *mcr*-4 gene; five swine manuscripts studied *mcr*-5 gene; one swine manuscript was about *mcr*-6, *mcr*-7, *mcr*-8, *mcr*-9 genes and one poultry study about *mcr*-10 gene was found. A great number of studies shows that meat-producing animals such as swine, bovine and poultry face a great danger by colistin exposition, and they are prone to spread the plasmid-mediated *mcr* genes.

The most detected gene is *mcr*-1 gene in *E. coli* from swine, bovine, poultry, and their products. The prevalence of colistin resistance in *Salmonella* spp. is usually low in healthy animals but depends on the ratio of serotypes that are inherently resistant to colistin. In addition, one review mentions the detection of *mcr*-1 in *A. baumannii*. The *mcr*-1, *mcr*-2 and *mcr*-3 are relatively common and widespread in meat producing animals. The identification of *mcr* variants in *Moraxella* species (*mcr-1* and *mcr-2*) and in *Aeromonas veronii* (*mcr-3*) proves that there are more organisms despite the Enterobacteriaceae that contribute to colistin resistance, and these might be responsible for the differences in prevalence observed. There is a great need to continually monitor, collect and present all relevant *mcr* gene resistance data globally, so as to be able to foresee public health threats and recommend the appropriate control measures to all stakeholders.

## Data Availability

No new data were created or analyzed in this study. Data sharing is not applicable to this article.
